# Impact of the COVID‐19 Outbreak on the Incidence of Autoimmune Encephalitis and Paraneoplastic Neurological Syndromes Associated Antibodies in Singapore

**DOI:** 10.1002/brb3.70630

**Published:** 2025-07-10

**Authors:** Rui Ling Rena Lau, Karine Su Shan Tay, Seyed Ehsan Saffari, Patricia Yut Wan Wong, Mei Ting Lim, Angelia Swee Hoon Koe, Jeanne May May Tan, Kok Pin Yong, Kevin Tan, Josiah Yui Huei Chai, Tianrong Yeo

**Affiliations:** ^1^ Duke‐NUS Medical School National University of Singapore Singapore Singapore; ^2^ Neuromuscular Laboratory National Neuroscience Institute Singapore Singapore; ^3^ Department of Neurology (Tan Tock Seng Hospital Campus) National Neuroscience Institute Singapore Singapore; ^4^ Department of Neurology (Singapore General Hospital Campus) National Neuroscience Institute Singapore Singapore; ^5^ Lee Kong Chian School of Medicine Nanyang Technological University Singapore Singapore

**Keywords:** antibodies, autoimmune encephalitis, COVID‐19, incidence, paraneoplastic neurological syndrome

## Abstract

**Background:**

Emerging evidence suggests a potential association between COVID‐19 and autoimmune encephalitis (AE). We aimed to evaluate the positivity rate of AE‐ and paraneoplastic neurological syndromes (PNS)‐associated antibodies in relation to COVID‐19.

**Methods:**

We investigated the frequency and incidence of AE‐ and PNS‐associated antibodies amongst clinical tests performed at the National Neuroscience Institute, Singapore, before and during the COVID‐19 pandemic. Antibodies against surface‐exposed antigens associated with AE were tested using cell‐based assays; antibodies against intracellular antigens in PNS were detected by immunoblot and tissue‐based assays.

**Results:**

A total of 87 of 4347 samples and 29 of 3393 samples tested for AE‐ and PNS‐associated antibodies, respectively, were positive. A spike in the incidence of AE‐associated antibodies was observed in 2020 at 4.92 (95% CI, 3.05–7.53) per 1,000,000 person‐years, coinciding with the first year of the COVID‐19 “pandemic outbreak.” The cumulative incidence in the “pre‐pandemic” period from 2017 to 2019 was 2.44 (95% CI, 1.66–3.46) per 1,000,000 person‐years (*p *= 0.034, vs. “pandemic outbreak”), and in the “mid to late pandemic” period from 2021 to 2023, this was 2.74 (95% CI, 1.91–3.82) per 1,000,000 person‐years (*p *= 0.086, vs. “pandemic outbreak”). The incidence of PNS‐associated antibodies was unaffected by the COVID‐19 pandemic.

**Conclusions:**

The increased incidence of AE‐associated antibodies during the COVID‐19 “pandemic outbreak” suggests a potential biological link. The subsequent decline in incidence in the “mid to late pandemic” period may be attributable to widespread vaccination and the emergence of new viral variants with less potential to induce autoimmunity. The incidence of PNS‐associated antibodies was stable throughout, reinforcing its primary association with malignancy.

## Graphical abstract

Emerging evidence suggests a potential association between COVID‐19 and autoimmune encephalitis (AE). We evaluated the positivity rate of AE‐ and paraneoplastic neurological syndromes (PNS)‐associated antibodies in relation to COVID‐19. A spike in the incidence of AE‐associated antibodies was observed during the COVID‐19 “pandemic outbreak” in 2020, compared to the “pre‐pandemic” period from 2017 to 2019 and to the “mid to late pandemic” period from 2021 to 2023. The incidence of PNS‐associated antibodies was unaffected by COVID‐19. The increased incidence of AE‐associated antibodies during the COVID‐19 “pandemic outbreak” suggests a potential biological link. The subsequent decline in incidence in the “mid to late pandemic” period may be attributable to widespread vaccination and the emergence of new viral variants with less potential to induce autoimmunity.

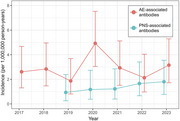

## 1 Introduction

There is growing recognition that SARS‐CoV‐2 infection may be associated with neurological complications, including autoimmune encephalitis (AE) (Valencia Sanchez et al. 2021). The biological rationale for this association comes from the observation that viruses can trigger AE, a prime example of which is *N*‐methyl‐d‐aspartate receptor (NMDAR) encephalitis following herpes simplex encephalitis (Leypoldt et al. 2013). Current evidence for the association of COVID‐19 with AE has been derived largely from case reports/series (Zambreanu et al. 2020; Shen et al. 2022); systematic reviews reveal that a subset of these cases harbor autoantibodies targeting surface‐exposed neuronal antigens that have been well‐described in AE (Nabizadeh et al. 2022; Xue et al. 2023). However, it is unclear if the incidence of AE (and indeed antibody‐positive AE) is higher during the pandemic compared to the pre‐pandemic era due to scarce epidemiological data.

The National Neuroscience Institute (NNI), Singapore clinical laboratory has performed autoantibody testing for AE and paraneoplastic neurological syndromes (PNS) since 2016 and 2018, respectively, receiving samples from healthcare facilities that provide neurological services to approximately three‐quarters of the population. We hypothesized that if COVID‐19 is associated with antibody‐positive AE, this would be reflected as a change in the frequency of antibody‐positive tests before and during the COVID‐19 pandemic. We also hypothesized that the frequency of PNS‐associated antibodies targeting intracellular onconeural antigens would be unchanged, given their primary association with malignancies rather than infective etiologies. To test these hypotheses, we investigated the frequency and incidence of AE‐ and PNS‐associated antibodies during the COVID‐19 pandemic compared to the pre‐pandemic period.

## 2 Methods

This was a retrospective observational study conducted at NNI with ethics board approval (CIRB 2024/2149).

Since August 2016, serum and cerebrospinal fluid (CSF) testing of AE‐associated antibodies (NMDAR, leucine‐rich glioma inactivated‐1 [LGI1], contactin‐associated protein‐like 2 [CASPR2], gamma‐aminobutyric acid receptor type B [GABA_b_R], alpha‐amino‐3‐hydroxy‐5‐methyl‐4‐isoxazolepropionic acid receptor 1/2 [AMPAR1/2], dipeptidyl‐peptidase‐like protein‐6 [DPPX]) has been performed using a commercial cell‐based assay (Euroimmun AG, Lubeck, Germany). For serum PNS‐associated antibodies (amphiphysin, collapsin response mediator protein 5 [CRMP5], glutamic acid decarboxylase 65 [GAD65], Hu, paraneoplastic Ma antigen 2 [PNMA2], recoverin, Ri, Sry‐like high mobility group box 1 [SOX1], titin, Tr, Yo, and zinc finger protein 4 [Zic4]), these were tested using immunoblot and tissue‐based assays (primate cerebellum and intestine) (Euroimmun AG, Lubeck, Germany) since August 2018. All tests were performed according to the manufacturer's instructions by experienced medical laboratory scientists and read by neurologists with expertise in the interpretation of these immunoassays.

To enhance the specificity of laboratory‐based diagnosis of AE and PNS in this study (i.e., to minimize false positivity), we applied robust inclusion criteria previously described in the literature and those from our clinical and laboratory experience. For NMDAR antibodies, only CSF‐positive samples (with or without seropositivity) were considered positive, as false negatives can occur with serum testing alone (Gresa‐Arribas et al. 2014). For CASPR2 antibodies, only samples with a titer ≥ 1:100 were considered positive given the possibility of non‐specific staining at 1:10, especially in serum, in line with the manufacturer's recommendation (Bien et al. 2017). For other AE‐associated antibodies, either serum and/or CSF positivity was considered positive. For PNS‐associated antibodies, positives were restricted to samples that demonstrated positivity on both immunoblot and tissue‐based assays due to the risk of false positivity when using immunoblot alone (Dechelotte et al. 2020). Antibodies against recoverin and titin detected on immunoblot were not taken to be positive, as a secondary confirmatory tissue‐based assay was unavailable for clinical purposes. When there were multiple samples from an individual, only the first sample was recorded. The study start date was specified as January 1, 2017, and January 1, 2019, for AE‐ and PNS‐associated antibodies, respectively (i.e., 5 months after initial introduction to allow test requests to stabilize), and the end date for data collection was December 31, 2023. The start of the COVID‐19 outbreak was defined as January 1, 2020.

Incidence rates and 95% confidence intervals (CI) were calculated using the exact method for the binomial distribution. Statistical comparison of incidence between time periods was performed using Poisson regression models with “person‐time duration” as an offset term with a logarithm link function. Tests for trends were calculated using the Poisson regression model, where “time” was included as a predictor. All statistical analyses were performed using R (R Foundation for Statistical Computing, Vienna, Austria). *p* values were two‐tailed, and significance was set at < 0.05.

## 3 Results

A total of 4347 serum and CSF samples were tested for AE‐associated antibodies, with an incremental number of samples tested year‐on‐year (except in 2021) (*p* value for trend < 0.0001). A total of 87 samples were positive: 43 NMDAR, 29 LGI1, 13 GABA_b_R, 1 CASPR2, and 1 DPPX. For PNS‐associated antibodies, 3393 serum samples were tested with an increasing number of tests performed in consecutive years (*p* value for trend < 0.0001). A total of 29 samples were positive: 9 GAD, 5 Yo, 4 Hu, 3 SOX1, 2 Zic4, 2 Ri, 1 Amphiphysin, 1 CRMP5, 1 PNMA2, and 1 Tr.

**FIGURE 1:**
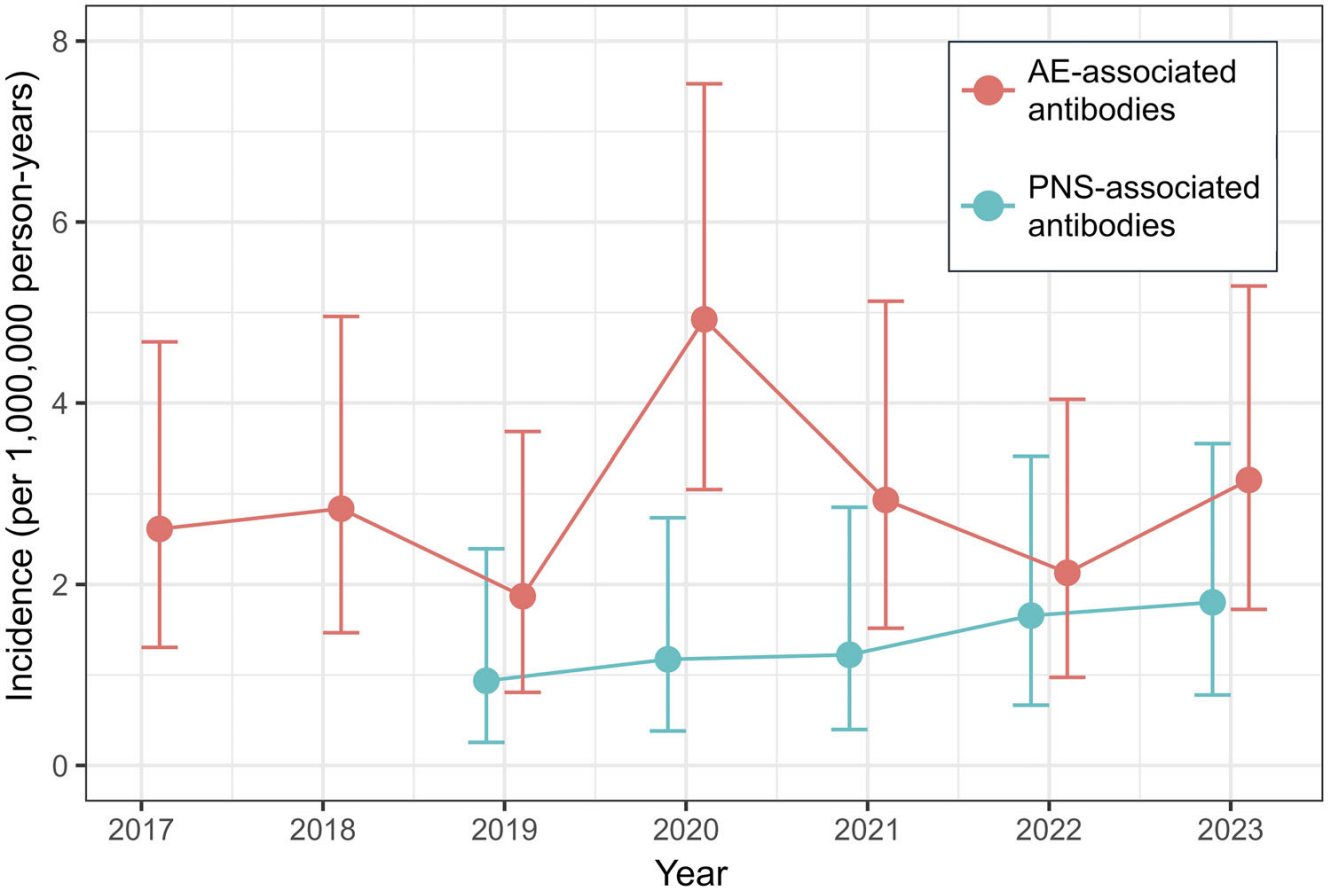
Incidence of autoimmune encephalitis‐ and paraneoplastic neurological syndromes‐associated antibodies before and during the COVID‐19 pandemic. Incidence of AE‐associated antibodies peaked in 2020 at 4.92 (95% CI, 3.05–7.53) per 1,000,000 person‐years, coinciding with the first year of the COVID‐19 "pandemic outbreak". The cumulative incidence in the "pre‐pandemic" period from 2017 to 2019 was 2.44 (95% CI, 1.66–3.46) per 1,000,000 person‐years (*p *= 0.034, vs. "pandemic outbreak"); the cumulative incidence in the "mid to late pandemic" period from 2021 to 2023 was 2.74 (95% CI, 1.91–3.82) per 1,000,000 person‐years (*p *= 0.086, vs. "pandemic outbreak"). Incidence of PNS‐associated antibodies showed no relation to the COVID‐19 pandemic. Error bars represent 95% CI.

The total number and frequency of positive AE‐associated antibodies were highest in 2020, coinciding with the first year of the COVID‐19 “pandemic outbreak” (Table 1). As there was an incremental number of tests performed over time (likely due to increased awareness of AE by clinicians), this could have led to a lower proportion of positive results (relative to all tests performed per annum) in the latter years of the study period. To address this, the incidence of AE‐associated antibodies was calculated per annum, using 75% of the Singaporean population for that year as a reference denominator (data from https://www.singstat.gov.sg/publications/population/population‐trends), as NNI receives samples from approximately three‐quarters of the populace. A spike in the incidence of AE‐associated antibodies was observed in 2020 at 4.92 (95% CI, 3.05–7.53) per 1,000,000 person‐years, coinciding with the COVID‐19 “pandemic outbreak” period (Table 1, Figure 1). The cumulative incidence in the “pre‐pandemic” period from 2017 to 2019 was 2.44 (95% CI, 1.66–3.46) per 1,000,000 person‐years (*p *= 0.034, vs. “pandemic outbreak”), and the cumulative incidence in the “mid to late pandemic” period from 2021 to 2023 was 2.74 (95% CI, 1.91–3.82) per 1,000,000 person‐years (*p *= 0.086, vs. “pandemic outbreak”).

**TABLE 1:** Frequency and incidence of autoimmune encephalitis‐ and paraneoplastic neurological syndromes‐associated antibodies per annum.

Year	Number of tests done ^a^, ^b^	Number of positive tests	Breakdown of positive tests	Positive rate (relative to the total number of tests per annum) (%)	Positive rate (relative to all positive tests) (%)	Incidence/1,000,000 person‐years (95% CI) ^c^, ^d^
**Autoimmune encephalitis‐associated antibodies**						
2017	381	11	4 NMDAR 4 LGI1 3 GABA_B_R	2.89	12.6	2.61 (1.30–4.68)
2018	513	12	8 NMDAR 4 LGI1	2.34	13.8	2.84 (1.47–4.68)
2019	530	8	6 NMDAR 1 LGI1 1 CASPR2	1.51	9.20	1.87 (0.807–3.68)
2020	666	21	9 NMDAR 7 LGI1 5 GABA_B_R	3.15	24.1	4.92 (3.05–3.68)
2021	650	12	5 NMDAR 5 LGI1 2 GABA_B_R	1.85	13.8	2.93 (1.52–5.12)
2022	786	9	4 NMDAR 3 LGI1 2 GABA_B_R	1.15	10.3	2.13 (0.973–4.04)
2023	821	14	7 NMDAR 5 LGI1 1 GABA_B_R 1 DPPX	1.71	16.1	3.15 (1.72–5.29)
Total	4347	87	—	—	—	—
**Paraneoplastic neurological syndromes‐associated antibodies**						
2019	483	4	2 GAD65 2 Yo	0.828	13.8	0.935 (0.255–2.39)
2020	615	5	2 Zic4 1 GAD65 1 Hu 1 SOX1	0.813	17.2	1.17 (0.381–2.74)
2021	692	5	1 Amphiphysin 1 GAD65 1 PNMA2 1 SOX1 1 Yo	0.723	17.2	1.22 (0.397–2.85)
2022	751	7	3 GAD65 2 Hu 1 CRMP5 1 Ri	0.932	24.1	1.66 (0.666–3.41)
2023	852	8	2 GAD65 2 Yo 1 Hu 1 Ri 1 SOX1 1 Tr	0.939	27.6	1.8 (0.778–3.55)
Total	3393	29	—	—	—	—

*Note*: Incidence is calculated based on 75% of the Singaporean population. The total population in Singapore was—2017: 5,612,300; 2018: 5,638,700; 2019: 5,703,600; 2020: 5,685,800; 2021: 5,453,600; 2022: 5,637,000; 2023: 5,917,600. Data from https://www.singstat.gov.sg/publications/population/population‐trends.

^a^ *p* value for trend (number of AE‐associated antibody tests performed from 2017 to 2023) < 0.0001.

^b^ *p* value for trend (number of PNS‐associated antibody tests performed from 2019 to 2023) < 0.0001.

^c^ *p* value for trend (incidence of positive AE‐associated antibody tests from 2017 to 2023) = 0.767.

^d^ *p* value for trend (incidence of positive PNS‐associated antibody tests from 2019 to 2023) = 0.214.

For PNS‐associated antibodies, a gradual increase in the number of positive tests from 2019 to 2023 was observed (corresponding to the incremental number of tests performed year‐on‐year, likely due to the increased awareness of PNS) (Table 1). PNS incidence was largely unaffected by the COVID‐19 pandemic, with a gradual increase in consecutive years from 0.94 (95% CI, 0.26–2.39) per 1,000,000 person‐years in 2019 to 1.80 (95% CI, 0.78–3.55) per 1,000,000 person‐years in 2023 (*p* value for trend = 0.214) (Table 1, Figure 1).

## 4 Discussion

While the pre‐pandemic incidence of AE‐associated antibodies in our study was comparable to previous reports (Hebert et al. 2020), we found a ∼two‐fold increase in 2020, which coincided with the COVID‐19 pandemic outbreak. This was unlikely to be attributable to higher testing volume, as the incidence in 2021 to 2023 decreased despite more samples being tested. Amongst the positive tests in 2020, NMDAR antibodies were the most common antibody type, followed by LGI1 and GABA_b_ antibodies. A significant increase in NMDAR encephalitis during the COVID‐19 pandemic was reported by the Barcelona group, although the overall positivity rate of neuronal‐surface antibodies did not change significantly (Arino et al. 2023). The Oxford group showed that the proportion of positive LGI1 antibody results was significantly reduced from March to June 2020, overlapping with the first UK pandemic “lockdown,” inferring that LGI1 antibody could potentially be triggered by infectious agents, including SARS‐CoV‐2 (Handel et al. 2023). The exact pathogenic mechanisms underlying the development of AE after SARS‐CoV‐2 infection remain to be fully elucidated. Proposed hypotheses include (1) molecular mimicry, whereby antibodies generated in response to the viral infection cross‐react with endogenous neural antigens, and (2) systemic hyperinflammatory response to the infection resulting in excessive inflammatory cytokines that disrupt the blood‐brain barrier and contribute directly to neuroinflammation with the subsequent development of AE (Vasilevska et al. 2024).

The decline in AE‐associated antibody incidence from 2021 to 2023 after the initial COVID‐19 pandemic outbreak is notable. This was observed despite a higher testing volume and a shorter period of “lockdown” measures (i.e., 6 weeks in 2021 vs. 8 weeks in 2020). We postulate that (1) the successful implementation of SARS‐CoV‐2 vaccinations (this began on December 30, 2020, and by October 27, 2021, 84% of the Singaporean population had completed two vaccine doses) resulted in fewer infections and reduced viral replication postinfection (Ministry of Health Singapore 2021; Puhach et al. 2022), hindering the potential to trigger an autoimmune response, and (2) the emergence of SARS‐CoV‐2 variants (which were more transmissible but resulted in milder infections) with attenuated potential to induce autoimmunity may have contributed to the decrease in incidence.

Our study has several limitations. The lack of clinical corroboration precludes definitive diagnosis of AE and PNS. It is also possible that some AE that occurred during the COVID‐19 pandemic were triggered by underlying tumors, and clinico‐serological confirmation of SARS‐CoV‐2 infection in AE antibody‐positive samples from 2020 to 2023 was unavailable; hence, the temporal association between COVID‐19 and AE antibody positivity could not be ascertained. We also acknowledge that our laboratory methods may risk false positive results; however, we believe this probability to be small due to the strict inclusion criteria applied. Furthermore, our testing methods have not changed before or during COVID‐19; hence, any change in the incidence of positive samples is unlikely to be attributable to false positivity. In conclusion, we demonstrated a spike in the incidence of AE‐associated antibodies during the initial COVID‐19 outbreak, suggesting a possible biological link. Our work underscores the importance of ongoing vigilance of the potential impact of COVID‐19 on the neurological system as COVID‐19 becomes endemic.

## Author Contributions

**Rui Ling Rena Lau**: conceptualization, investigation, writing—original draft, methodology, formal analysis. **Karine Su Shan Tay**: methodology, data curation, investigation, project administration, writing—review and editing. **Seyed Ehsan Saffari**: formal analysis, visualization, writing—review and editing. **Patricia Yut Wan Wong**: investigation, writing—review and editing. **Mei Ting Lim**: investigation, writing—review and editing. **Angelia Swee Hoon Koe**: investigation, writing—review and editing. **Jeanne May May Tan**: investigation, writing—review and editing. **Kok Pin Yong**: investigation, writing—review and editing. **Kevin Tan**: investigation, writing—review and editing. **Josiah Yui Huei Chai**: investigation, writing—review and editing, resources. **Tianrong Yeo**: conceptualization, methodology, investigation, formal analysis, supervision, writing—original draft, writing—review and editing.

## Ethics Statement

The study complies with the Declaration of Helsinki's ethical standards. Waiver of consent was sought and approved by the SingHealth ethics board (CIRB 2024/2149). The information presented pertains to professional knowledge and experiences in a specific field; no personal or sensitive data that would pose ethical risks were collected in this study.

## Conflicts of Interest

Jeanne May May Tan has received honoraria from Merck for speaker's fees and research grants from the National Medical Research Council (Singapore) and National Neuroscience Institute (Singapore). Kevin Tan has received travel grants and compensation from Novartis, Merck, Sanofi, Eisai, Roche, and Terumo BCT for consulting services and education activities. Tianrong Yeo has received honoraria from ASNA, Edanz Pharma, Euroimmun AG, Merck, Novartis, Roche, and Terumo BCT for consulting services and speaker's fees and research grants from the National Medical Research Council (Singapore), AstraZeneca, and Roche. He has also received travel grants and awards from PACTRIMS, ACTRIMS, ECTRIMS, Orebro University, UCB, and Merck. The other authors declare no conflicts of interest.

## Peer Review

The peer review history for this article is available at https://publons.com/publon/10.1002/brb3.70630

## Data Availability

The data that support the findings of this study are available from the corresponding author on reasonable request.
